# HIV Infects Bronchial Epithelium and Suppresses Components of the Mucociliary Clearance Apparatus

**DOI:** 10.1371/journal.pone.0169161

**Published:** 2017-01-06

**Authors:** S. Chinnapaiyan, T. Parira, R. Dutta, M. Agudelo, A. Morris, M. Nair, H. J. Unwalla

**Affiliations:** 1 Department of Immunology, Institute of Neuroimmune Pharmacology, Herbert Wertheim College of Medicine, Florida International University. Miami, Florida, United States of America; 2 Division of Pulmonary, Allergy, and Critical Care Medicine, Department of Medicine, University of Pittsburgh, Pittsburgh, Pennsylvania, United States of America; Beckman Research Institute, UNITED STATES

## Abstract

Recurrent lung infections and pneumonia are emerging as significant comorbidities in the HIV-infected population in the era of combination antiretroviral therapy (cART). HIV infection has been reported to suppress nasal mucociliary clearance (MCC). Since the primary components driving nasal MCC and bronchial MCC are identical, it is possible that bronchial MCC is affected as well. Effective MCC requires optimal ciliary beating which depends on the maintenance of the airway surface liquid (ASL), a function of cystic fibrosis transmembrane conductance regulator (CFTR) activity and the integrity of the signaling mechanism that regulates ciliary beating and fluid secretion. Impairment of either component of the MCC apparatus can compromise its efficacy and promote microbial colonization. We demonstrate that primary bronchial epithelium expresses HIV receptor CD4 and co-receptors CCR5 and CXCR4 and can be infected by both R5 and X4 tropic strains of HIV. We show that HIV Tat suppresses CFTR biogenesis and function in primary bronchial epithelial cells by a pathway involving TGF-β signaling. HIV infection also interferes with bronchial epithelial cell differentiation and suppresses ciliogenesis. These findings suggest that HIV infection suppresses tracheobronchial mucociliary clearance and this may predispose HIV-infected patients to recurrent lung infections, pneumonia and chronic bronchitis.

## Introduction

MCC is a primary innate defense mechanism of mammalian airways that protects the host from the noxious effects of airborne pathogens, pollutants and allergens [1]. The MCC apparatus consists of the cilia, a protective mucus layer, and a periciliary Airway surface liquid (ASL) layer to optimize ciliary beating [2]. Abnormalities in any compartment of the mucociliary system can compromise mucus clearance leading to mucus impaction and consequently, chronic bacterial infection [3–5]. The height of the ASL layer lining the airway surfaces is crucial for mediating MCC rates [6] and is tightly regulated by CFTR [7]. CFTR dysfunction can have a pronounced effect on ASL depth as well as ciliary beating and can contribute directly to microbial colonization. Bacterial pneumonia and COPD are the most prevalent lung comorbidities in people living with HIV [8,9]. Lung infections are exacerbated in HIV-infected smokers [10,11] and this could be due to the ability of cigarette smoke to independently attenuate both CFTR function [12,13] and ciliary beating. HIV-infected patients show abnormalities in their nasal MCC apparatus [14,15]. However, nasal Cl^-^ efflux and CBF is often measured as a barometer of overall airway MCC health [16–18]. Hence it is possible that tracheobronchial mucociliary clearance is affected as well. HIV has also been recovered from cell-free bronchoalveolar lavage fluid [19] suggesting that it can directly mediate its effects in the airway.

In our earlier report we have demonstrated that TGF-β signaling and cigarette smoke (via TGF-β signaling) suppresses CFTR biogenesis and function [12]. HIV Tat can induce TGF-β signaling in different cells types [20–22] possibly by binding to a Tat responsive element in the TGF-β1 promoter [22,23]. In this study, we will show that HIV Tat also suppresses CFTR biogenesis and function via TGF-beta signaling. Tat can be expressed and secreted from infected cells even in presence of cART due to a protein transduction domain. Secreted Tat can be taken up by bystander cells where it can have pleotropic effects. [24,25]. Considering reports that HIV can productively [26] or non-productively [27] infect epithelia, we will show that primary human bronchial epithelium expresses canonical HIV receptors and can be infected with HIV. Its effects on the Mucociliary clearance apparatus will be discussed.

## Results

### HIV Tat suppresses CFTR biogenesis and function via TGF-β signaling

**Fig 1:**
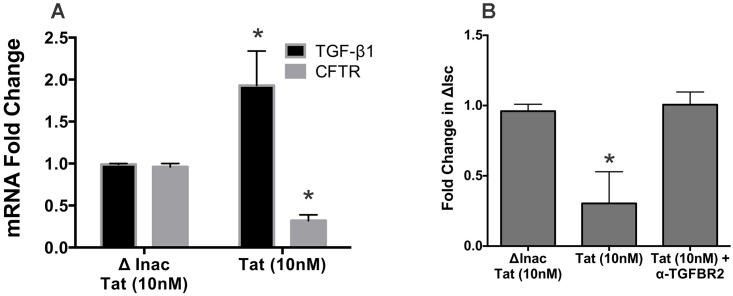
HIV Tat suppresses CFTR biogenesis and function. **Panel A**: HIV Tat induces expression of TGF-β1 mRNA. NHBE redifferentiated at the ALI were treated with recombinant Tat (10nM) or heat inactivated Tat (as control) apically and basolaterally. After 24 hours, Medium was changed and replaced with fresh medium containing Tat. Forty-eight hours post- Tat treatment, total RNA was isolated and TGF-β1 and CFTR mRNA levels were quantitated by qRT-PCR. HIV Tat significantly induces TGF-β1 mRNA expression with a concomitant suppression of CFTR mRNA. **Panel B**: HIV Tat suppresses CFTR function via TGF-β signaling. NHBE ALI cultures grown on snapwells were treated to HIV Tat as mentioned. Separately, cells were treated with anti-TGFBR2 antibody (added 3 hours before treatment apically and basolaterally; 25 μg/ml) and retained for the remainder of the experiment. Cells were mounted in Ussing chambers and Cl^-^ efflux in response to albuterol addition was determined in the presence of amiloride. To ensure that change in short circuit current (ΔI_SC_) is CFTR specific, Ussing chamber experiments were terminated by addition of CFTRinh172 (20 μM). HIV Tat decreases CFTR function. Anti-TGFBR2 neutralizing antibody caused a complete restoration of CFTR function in Tat treated cells. n = NHBE ALI cultures from 3 different lungs * = significant (p < 0.05).

NHBE ALI cultures were treated with Tat (heat inactivated Tat as control). Several groups have reported HIV Tat in the serum in the sub-nanomolar range [28,29] and Tat may also bound to endogenous glycosaminoglycans and heparin sulfates in vivo which cannot be measured [30] We have used a concentration of Tat at a dose found in the serum and also used by others studies [31–34]. After forty-eight hours, cells were lysed and total RNA was extracted and analyzed for changes in TGF-β and CFTR mRNA by qRT-PCR. Tat increased TGF-β1 mRNA levels with a concomitant decrease in CFTR mRNA levels (Fig 1A). To determine if decrease in CFTR mRNA translates to a corresponding decrease in CFTR function, NHBE redifferentiated at the ALI were similarly treated with Tat, forty-eight hours post-initial treatment, cells were mounted in Ussing chamber and CFTR specific short circuit current was determined by adding albuterol as reported [12]. Separately, to determine if Tat exerts its effect via TGF-β signaling, another set of Tat treated NHBE ALI cultures were pretreated with anti-TGFBR2 before Tat addition. As seen in Fig 1B, HIV Tat mediated suppression of CFTR mRNA leads to a concomitant suppression of CFTR function. Anti-TGFBR2 antibody was able to rescue HIV Tat mediated suppression of CFTR function suggesting that HIV Tat mediates suppresses CFTR via TGF-β signaling. The extent for mRNA suppression with Tat and TGF-β (previous report [12]) directly relates to the extent of suppression of CFTR function suggesting that suppression of CFTR biogenesis is responsible for suppression of CFTR function.

### Bronchial epithelial cells express canonical HIV receptors and can be infected with HIV

**Fig 2:**
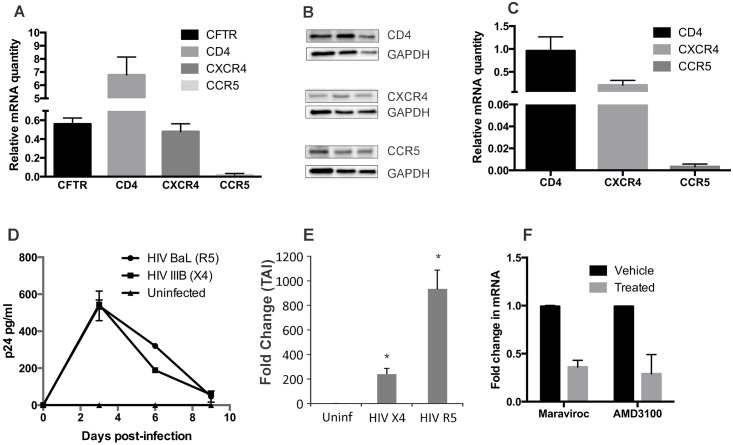
NHBE redifferentiated at the ALI express HIV receptors and co-receptors and can support both R5 and X4-tropic infection. **Panel A**: We analyzed expression of CD4, CCR5 and CXCR4 in NHBE by qRT-PCR. Total RNA was isolated from NHBE ALI cultures and expression of CD4 (Applied Biosystems # HS01058407-m1), CXCR4 (Applied Biosystems # HS00607978-s1) and CCR5 (Applied Biosystems # HS99999149-s1) was determined by qRT-PCR using Taqman probes. CFTR (Applied Biosystems # HS00357011-m1) expression was determined for comparison. NHBE ALI cultures express all canonical HIV receptors. **Panel B**: Western blot analysis from three different lungs demonstrates that NHBE ALI cultures show comparable expression of all HIV receptors. While RNA levels were significantly lower for CCR5 than CD4 and CXCR4, protein levels were comparable with CD4 and CXCR4. This could be due to inherent CCR5 mRNA instability due to a pseudoknot structure in the mRNA that promotes non-sense mediated decay [35]. **Panel C**: To determine if bronchial epithelial cells express HIV receptors and co-receptors in vivo, Bronchial brushings were analyzed for expression of CD4, CCR5 and CXCR4 by qRT-PCR. Expression patterns of the three receptors were similar to that observed in our ex vivo model with maximal expression of CD4 followed by CXCR4 and significantly lower CCR5 expression validating the physiological relevance of our ex vivo NHBE ALI culture model. **Panel D and E**: NHBE ALI cultures can be infected with both R5 and X4-tropic strains of HIV. NHBE cultures redifferentiated at the Air-Liquid Interface were infected apically and basolaterally with either HIV IIIB (X4-tropic) or HIV BaL (R5-tropic) strains. After 16 hours cells were washed apically and basolaterally with PBS four times to remove any residual input virus. The fourth wash was collected for p24 analysis and measured as Day 0 to confirm that all input virus had been removed. No p24 was detected in the 4^th^ wash (Day 0) confirming that all input virus had been removed. We observe an initial spike in p24 output on Day 3 for both HIV IIIB and HIV BaL infections that declines gradually till Day 9 (panel D). Experiments were terminated and total RNA was isolated and cell associated HIV RNA was quantitated by qRT-PCR using Taqman probe (Applied Biosystems # PA03453409-s1). NHBE cells demonstrate cell associated viral RNA for both R5 and X4-tropic infections (panel E). **Panel F**: Another set of NHBE ALI cultures similarly infected in presence of with maraviroc (For HIV BaL infection) or AMD3100 (for HIV IIIB infection) which was retained for the remainder of the experiment. Maravoiroc was able to block infection of NHBE ALI cultures by the R5-tropic strain HIV BaL. Likewise, AMD3100 was able to block infection by the X4-tropic strain HIV IIIB. n = NHBE ALI cultures from 3 different lungs * = significant (p < 0.05).

Expression of HIV receptors and co-receptors has been demonstrated in other epithelial cells [26] which can be productively [26] or non-productively [27] infected. We first analyzed expression of CD4, CCR5 and CXCR4 in NHBE by qRT-PCR (CFTR used for comparison) and western blot (3 different lungs). As seen in Fig 2A and 2B, NHBE redifferentiated at the ALI expressed all canonical HIV receptors. While detectable RNA levels were lower for CCR5, protein levels were comparable with CD4 and CXCR4. This difference in detectable RNA and protein levels for CCR5 could be due to inherent CCR5 mRNA instability due to a pseudoknot structure in the mRNA that promotes non-sense mediated decay [35] and tightly regulates CCR5 expression. To determine if we observe similar expression in vivo, bronchial brushings from HIV-infected patients was analyzed for expression of HIV receptors and co-receptors. We observed a similar pattern of expression in bronchial brushings with maximal expression observed for CD4 followed by CXCR4 and CCR5 (Fig 2C).

Next, we determined if HIV could infect NHBE redifferentiated at the ALI. Fully differentiated NHBE ALI cultures were infected basolaterally and apically with the X4-tropic HIV (HIV IIIB) or the prototypic R5-tropic HIV (HIV BaL) for 16 hours. At designated time points, culture supernatants were collected and analyzed for p24 production by ELISA. To exclude measuring any p24 in the supernatant from residual input virus, sixteen hours post-infection, cells were washed four times with PBS apically and basolaterally and the final wash (Day 0) was collected and analyzed for p24. As seen in Fig 2D, no detectable p24 is observed on day 0 after washes suggesting that p24 levels on subsequent time points could only have been due to infection of NHBE cells. Maximal p24 production is observed on day 3 with both X4-tropic and R5-tropic HIV that declines progressively until Day 9. On Day 9, total RNA was isolated from these cells and analyzed for HIV cell-associated RNA using Taqman probe (life technologies cat # PA03453409_s1). As seen in Fig 2E we were able to detect HIV RNA from NHBE ALI cultures infected with both HIV IIIB and HIV BaL. Another set of NHBE ALI cultures infected similarly were pretreated with R5-inhibitor maraviros or X4-inhibitor AMD3100. The inhibitors were retained for the remainder of the experiment by adding them to fresh medium during media changes. Fig 2F shows that both Maraviroc and AMD3100 block infection of NHBE ALI cultures by their respective strains. Culture conditions established for primary bronchial epithelial cells differentiation and maintenance prevent growth of immune cells (Scott Randell; University of North Carolina, Chapel Hill; personal communication). To confirm that our primary human bronchial epithelium was devoid of any contaminating immune cells, NHBE ALI cultures were stained for the pan-leukocyte antigen marker CD45 and analyzed by flowcytometry. NHBE cells were found to be CD45 negative confirming that the primary bronchial cells do not have any contaminating immune cells (S1 Fig). These data suggest that p24 output and viral RNA are due to infection of primary bronchial epithelial cells.

### HIV establishes long-term restricted infection in primary bronchial epithelial cells

**Fig 3:**
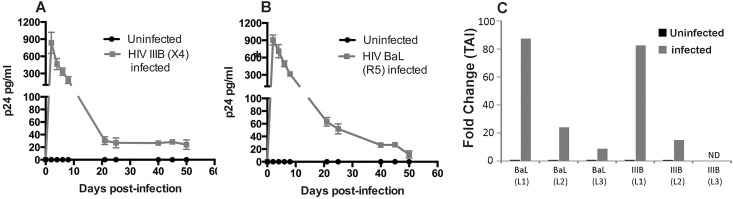
Long-term HIV infection in NHBE ALI cultures. **Panel A and B**: NHBE cultures redifferentiated at the Air-Liquid Interface from three different lungs were infected apically and basolaterally with either HIV IIIB (X4-tropic) or HIV BaL (R5-tropic) strains. After 16 hours cells were washed apically and basolaterally with PBS four times to remove any residual input virus. The fourth wash was collected for p24 analysis and measured as Day 0 to confirm that all input virus had been removed. At designated time points, culture supernatants were collected and measured for p24 using the p24 ELISA kit. No p24 was detected in the 4^th^ wash (Day 0) confirming that all input virus had been removed. Maximal p24 is detected on Day 2 and then decreases gradually. Detectable p24 is observed up to Day 50 post-infection. Experiments were terminated on Day 50 due to excessive cell death. **Panel C**: qRT-PCR analysis of cell associated viral RNA from three independent lungs that were infected with HIV IIIB and HIV BaL. HIV RNA is detected in all cultures infected with HIV BaL and two of three cultures infected with HIV IIIB. n = NHBE ALI cultures from 3 different lungs * = significant (p < 0.05). ND = Not Done (due to cell death).

Next we tried to determine if HIV was capable of establishing long-term infection in NHBE ALI cultures. We infected NHBE cultures redifferentiated at the ALI with HIV IIIB and HIV BaL as described before. Culture supernatants were collected for p24 analysis for an initial eight days and then over long-term at 21, 25, 41, 45 and 50 days. Experiments were terminated on Day 50 due to excessive cell death observed in infected NHBE ALI cultures. Total RNA was recovered and analyzed for HIV RNA by qRT-PCR. As seen in Fig 3A and 3B, a spike in p24 is observed that declines by day 8. However, stable levels of detectable p24 are observed up to 50 days post-infection. qRT-PCR for HIV RNA analysis 50-days post-infection, shows cell-associated viral RNA in all HIV BaL infected and two out of three HIV IIIB infected NHBE ALI cultures (Fig 3C). The single HIV IIIB infected culture in which we were not able to detect cell associated viral RNA was due to very low levels of RNA that were recovered from this culture due to excessive cell death.

### HIV infects NHBE cells and undergoes reverse transcription to generate proviral DNA

One possibility that would account for a non-productive infection [36] is if the proviral DNA fails to integrate and is maintained in the episomal form [37]. Episomal forms of proviral DNA have been shown to be transcriptionally active [38,39]. Nef expressed from unintegrated HIV DNA has been shown to downregulate HIV receptors [40]. We first tried to determine if we could detect integrated or episomal forms of HIV DNA. NHBE ALI cultures were infected as described in methods. Twelve days post-infection genomic DNA was isolated and presence of integrated HIV was detected using the 2-step PCR approach described by Brussel and Sonigo [41]. This approach specifically detects only integrated viral DNA. Simultaneously another set of similarly infected cells were tested for presence of episomal viral DNA using the approach described by Buzon et. al. [42]. As seen in Table 1 we detected both integrated and episomal HIV DNA.

**Table 1:** HIV infects NHBE cells and undergoes reverse transcription to generate proviral DNA.

**INTEGRATED**	**Ct**	**Copies/10μL**
HIV IIIB L1	36.98	139
HIV IIIB L2	38.36	67
HIV IIIB L3	37.19	143
HIV BaL L1	38.93	45
HIV BaL L2	38.06	81
HIV BaL L3	37.19	130
**EPISOMAL**		
HIV IIIB L1	33.25	1182
HIV IIIB L2	32.61	1706
HIV IIIB L3	ND	ND
HIV BaL L1	41.22	12
HIV BaL L2	46.78	<3
HIV BaL L3	34.67	523

NHBE cultures redifferentiated at the Air-Liquid Interface from three different lungs were infected with HIV IIIB or HIV BaL strain as described before. Six days post-infection, genomic DNA was isolated and analyzed for integrated proviral DNA using the 2-step PCR protocol described by Brussel and Sonigo [41]. NHBE ALI cultures infected with HIV IIIB and HIV BaL demonstrate the presence of integrated proviral DNA. For episomal HIV proviral DNA, NHBE cultures redifferentiated at the Air-Liquid Interface from three different lungs were infected with HIV IIIB or HIV BaL strain as described before. Six days post-infection, DNA was extracted using the Qiagen Plasmid Miniprep kit according to the manufacturer’s protocol. Extracted DNA was concentrated and analyzed by qPCR analysis using the HIV specific Taqman probe (Applied Biosystems # PA03453409-s1). Episomal HIV DNA was detected in two out of three HIV IIIB and HIV BaL infected NHBE ALI cultures. ND = Not detected. L = Lung.

**Fig 4:**
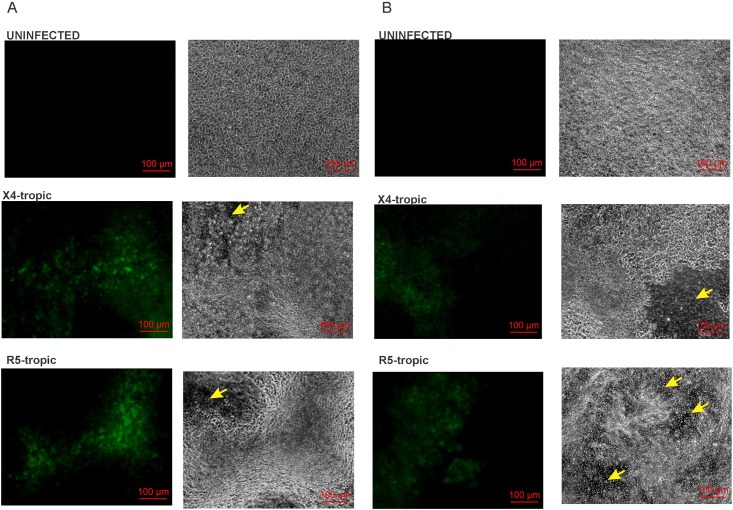
A fluorescent HIV reporter demonstrates HIV undergoes reverse transcription and expresses viral genes in NHBE ALI cultures. A fluorescent HIV vector (RGH-WT) reported by Dahabieh et. al. [43] was packaged in HEK 293T cells using either R5-tropic env (pBal.01) or X4 tropic envelope (pHXB2 env). 10ngs p24 equivalent of both viruses was used to infect NHBE ALI cultures. On Day 6 and 12 cells were visualized using a Zeiss fluorescence microscope with high resolution Axiocam 506 mono microscope camera (Zeiss, Germany). GFP expression is observed in NHBE ALI cultures infected with RGH-WT virus enveloped with R5 envelope and X4 envelope. Expression of GFP was observed as distinct foci in the epithelium. Phase contrast microscope images demonstrate some cell death (yellow arrows) by Day 6 and significant cell death and disruption of epithelial barrier on Day 12 post-infection. Since this virus is env^-^, cytotoxicity and disruption of epithelial layer could only be due to death of infected NHBE cells or a bystander effect of viral proteins (other than env) secreted by infected cells.

To confirm that HIV undergoes reverse transcription followed by gene expression, NHBE cells redifferentiated at the ALI were infected with the fluorescent RGH-WT HIV reporter reported by Dahabieh et. al. [43]. This virus expresses EGFP under the control of HIV LTR and mCherry under the control of a CMV promoter inserted in the env ORF. The virus is env^-^ and can be pseudo-typed with any envelope. Due to a lack of Env expression, it undergoes only a single round of infection. RGH-WT was enveloped with either R5-tropic env (pBaL.01) or X4-tropic envelope (pHXB2 env) in HEK 293T. 10ngs p24 equivalent of both viruses was used to infect NHBE ALI cultures and infection was allowed to proceed for 12 days. Even though the virus expresses both GFP and mCherry we decided to focus on GFP expression alone as this is directly under the control of HIV LTR and would be more representative of LTR mediated transcription. Moreover CMV promoter is not ideal for bronchial epithelial cells as it is rapidly silenced in these cells [44–46]. As seen in Fig 4, GFP expression is observed in NHBE ALI cultures infected with RGH-WT virus enveloped with R5 and X4 envelope on Days 6 and 12. On Day 12 post-infection, significant cell death leading to disruption of epithelial barrier was observed in infected NHBE cells (Fig 4B). These data indicate that HIV RNA is reverse transcribed upon entry into NHBE ALI cultures to proviral DNA and expresses GFP from the HIV LTR. Since the RGH-WT virus is incapable of productive infection, any cell death could only be due to a direct consequence of the initial infection or a bystander effect of viral proteins secreted by these cells.

### HIV-infected NHBE ALI cultures demonstrate decreased levels of HIV receptor expression and secrete functional Tat

**Fig 5:**
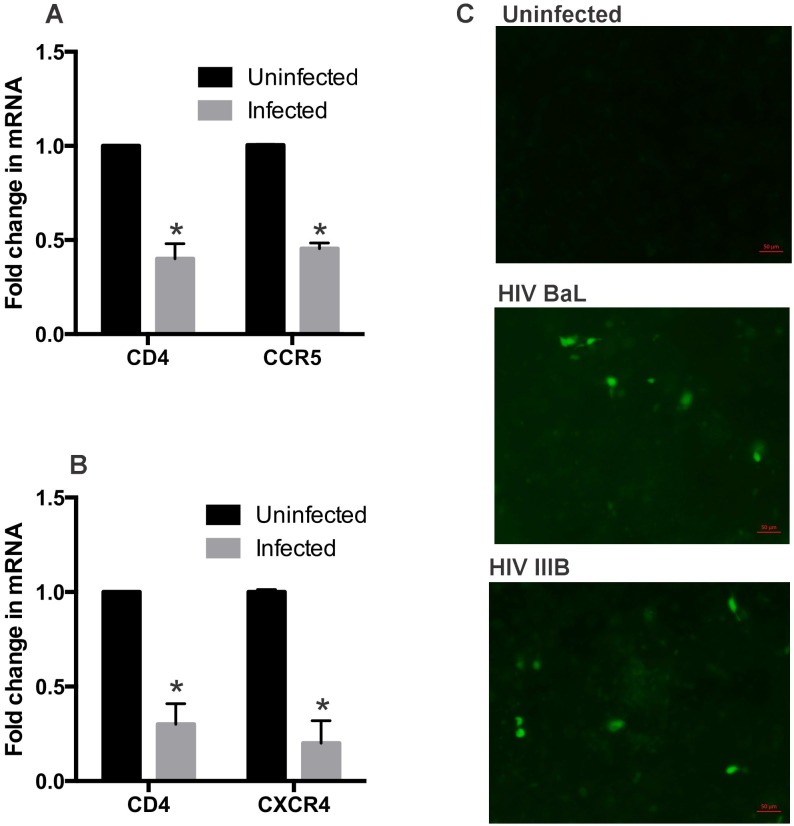
HIV-infected NHBE ALI cultures shows reduced levels of HIV receptors and secrete functional Tat. **Panel A**: NHBE ALI cultures redifferentiated at the Air-Liquid Interface were infected with HIV BaL strain as described in Fig 2. Nine days post-infection, total RNA was isolated and expression of CD4 and CCR5 was determined by qRT-PCR. HIV BaL infected NHBE ALI cultures show decreased CD4 and CCR5 expression. **Panel B**: NHBE ALI cultures redifferentiated at the Air-Liquid Interface were infected with HIV IIIB strain as described in Fig 2. Nine days post-infection, total RNA was isolated and expression of CD4 and CXCR4 was determined by qRT-PCR. HIV IIIB infected NHBE ALI cultures show decreased CD4 and CXCR4 expression. **Panel C**: Infected NHBE ALI cultures secrete functionally active Tat. Nine day old infected NHBE ALI cultures grown on transwells were placed on top of HEK 293 cells transiently transfected with LTRshGFPpA plasmid reported by us earlier [47]. In this construct GFP expression is controlled by HIV LTR such that GFP expression is only observed in presence of Tat. HEK 293 cells co-cultured with uninfected NHBE cells were used as controls. 24 hours post- co-culture, HEK 293 cells were visualized by fluorescence microscopy for EGFP expression. HEK-293 cells co-cultured with infected NHBE cells demonstrate EGFP positive cells suggesting that HIV Tat is secreted in the medium and this can transactivate LTR in co-cultured HEK 293 cells.

We determined if cell death due to infection manifests as decreased levels of HIV receptors in the NHBE ALI population. Total RNA from NHBE ALI cultures infected for 9 days by HIV BaL or HIV IIIB was analyzed for CD4, CCR5 and CXCR4 gene expression. Infection with both HIV BaL (Fig 5A) and HIV IIIB (Fig 5B) strains showed decreased mRNA levels of CD4 and their corresponding co-receptors. Decreased mRNA levels for cognate HIV receptors suggest a purging of cells that can be infected with HIV from the NHBE ALI cultures. Next we tried to determine if infected NHBE cells secrete Tat and if this can affect bystander cells. 9-day old infected NHBE ALI cultures grown on transwells were placed over HEK-293 cells transiently transfected with LTRshGFPpA plasmid reported by us earlier [47]. In this construct GFP expression is only observed in presence of Tat. HEK-293 cells co-cultured with uninfected NHBE cells were used as control. As seen in Fig 5C we observed GFP expression in cells co-cultured with NHBE ALI cultures infected with both HIV BaL and HIV IIIB suggesting that Tat is secreted by infected NHBE cells and activates LTR controlled GFP expression in co-cultured LTRshGFPpA transfected HEK-293 cells. These data further confirm viral entry, reverse transcription and gene expression in NHBE ALI cultures.

### HIV infects undifferentiated NHBE cells and interferes with ciliogenesis

**Fig 6:**
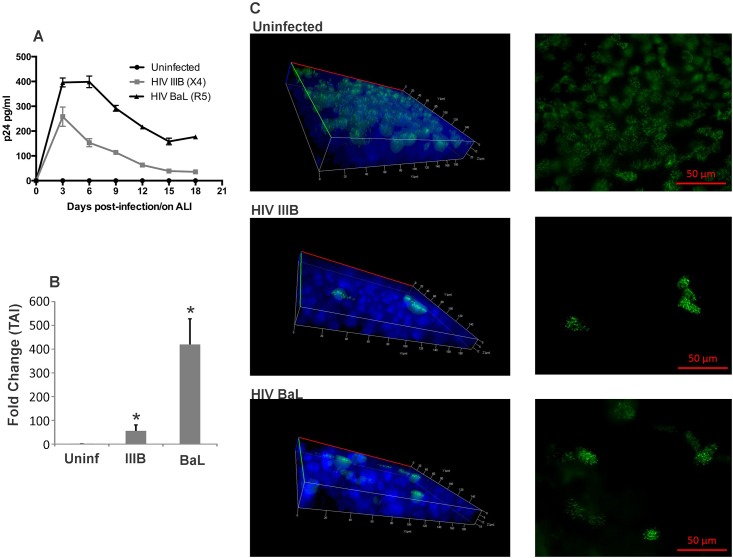
HIV infects undifferentiated NHBE cells and inhibits ciliogenesis. **Panel A**: NHBE ALI cultures were infected as described before on the day they were placed under Air-liquid culture conditions. Uninfected lung matched cultures were similarly differentiated as controls. At designated time points, culture supernatants were collected and analyzed for p24. Undifferentiated NHBE ALI cultures can support HIV infection. **Panel B**: On day 18 when ciliogenesis was observed in Lung-matched uninfected controls, experiments were terminated and total RNA was analyzed by qRT-PCR for presence of HIV RNA. HIV RNA was detected in both HIV IIIB and HIV BaL infected NHBE cells. n = NHBE ALI cultures from 3 different lungs. * = significant (p < 0.05). **Panel C**: Another set of cells were infected similarly was On Day 18 post-infection when ciliogenesis was observed in lung matched uninfected controls, Experiments were terminated, cells were fixed and stained for cilia as described by us earlier [50] and visualized using a Zeiss fluorescence microscope with high resolution Axiocam 506 mono microscope camera (Zeiss, Germany). HIV infection of NHBE ALI cultures impairs epithelial layer formation and interferes with ciliogenesis. Primary bronchial epithelium is pseudostratified. In a healthy well-differentiated epithelium, all ciliated cells will not be in the same plane. Hence some cilia are sharply focused in a particular plane while others, out of focus appear blurred. In infected cells, we do not observe the formation of the pseudostratified epithelium and the epithelial integrity is also impaired hence ciliated cells appear in a single plane.

Efficient MCC requires both CFTR function to provide optimal ASL depth and ciliary function. Since HIV Tat can induce TGF-β signaling in NHBE ALI cultures and TGF-β can alter miRNA homeostasis [48,49]. we determined if HIV can infect undifferentiated NHBE cells and inhibit ciliogenesis. NHBE cells were exposed to air to initiate differentiation and were infected with HIV IIIB or BaL. At designated time points, culture supernatant was collected and analyzed for p24 levels. Lung matched uninfected cultures were allowed to differentiate as controls. Experiments were terminated at Day 18 once adequate ciliogenesis was observed in uninfected controls. Fig 6A shows that HIV infects undifferentiated NHBE cells. Detectable p24 is observed up to 18 days post-infection. Total RNA isolated 18 days post-infection demonstrates the presence of cell associated HIV RNA. Another set of similarly infected cells were used to determine if HIV infection affects differentiation. On day 18 once adequate ciliogenesis was observed in uninfected controls, cells were fixed and stained for cilia as described by us earlier [50]. As seen in Fig 6 the thickness and integrity of the redifferentiated bronchial epithelium is greatly diminished in cells infected with HIV. In areas where the epithelium had formed, ciliogenesis was greatly diminished. These data indicate that HIV also suppresses ciliogenesis by infecting undifferentiated cells.

## Discussion

In this manuscript, We demonstrate for the first time that HIV infects both differentiated and undifferentiated primary bronchial epithelial cells. Upon infection, the viral RNA undergoes reverse transcription to express viral proteins Tat and p24. Tat suppresses CFTR biogenesis and function via TGF-β signaling. Infection of differentiating NHBE ALI cultures leads to suppression of ciliogenesis. Hence HIV infection can suppress ASL depth maintenance while at the same time decreasing ciliogenesis. People living with HIV show abnormalities in their nasal MCC apparatus [14,15]. However, nasal Cl^-^ efflux and CBF is often measured as a barometer of overall airway MCC health [16–18]. Our data suggest that infection of primary bronchial epithelium could be responsible. Effective MCC requires the combined action of ASL depth maintenance and ciliary beating. In normal conditions, apical nucleotides (ATP and its metabolites) are important for hydrating airway surfaces (e.g. see [51]). ATP binds to purinergic G-protein coupled receptors, leading to activation of Ca^2+^ dependent Cl^-^ channels and CFTR. Normal mucociliary function fails when the capabilities of the cAMP and ATP/Ca^2+^-mediated apical Cl^-^ efflux fail, leading to airway surface dehydration (e.g. see [52]). Since optimal ASL depth allows optimal ciliary beating any ASL dysregulation will also manifest as impaired ciliary beating. Likewise suppression of ciliogenesis or inhibition of signaling mechanisms that regulate ciliary beating can affect MCC as well. Attenuation of MCC leads to mucus impaction. The accumulated mucus entraps bacteria and promotes chronic bacterial infection [3]. TGF-β signaling upregulated in smoker and in chronic airway diseases suppresses CFTR biogenesis and function [12]. This could explain recurrent lung infections in people living with HIV.

We first looked at the effects of HIV Tat given reports that HIV Tat induces TGF-β1 expression in a number of cell types [20–22]. Expression of HIV Tat cannot be suppressed by anti-retrovirals [53–56]. Its protein transduction domain allows its secretion by infected cells and uptake by bystander cells where it mediates pleotropic effects [30,57–59]. Circulating Tat in the serum of HIV patients can reach nanomolar concentrations [28,29]. We used a dose that correlates with Tat found in the serum and also used in other studies [29,31–34]. Our observations demonstrate that HIV Tat suppresses CFTR biogenesis and function. A neutralizing antibody to TGFBR2 rescued the effect of HIV Tat on CFTR function suggesting that Tat mediates its effects on CFTR via TGF-β signaling.

Our results demonstrate that primary human bronchial epithelial cultures redifferentiated at the ALI express canonical HIV receptors CD4, CCR5 and CXCR4 and can be infected with both R5-tropic and X4-tropic HIV. This infection could be blocked by respective CCR5 and CXCR4 inhibitors. Gundavarapu et. al.[60] have demonstrated expression of CXCR4 but not CCR5 mRNA in NHBE cells by qRT-PCR. Although we were able to detect very low levels of CCR5 mRNA in our cultures, western blot analysis showed that CCR5 protein levels were comparable with CD4 and CXCR4. This could be due to inherent CCR5 mRNA instability due to a pseudoknot structure that functions in combination with microRNAs to promote non-sense mediated decay [35]. It is possible that expression of CCR5 is tightly regulated in bronchial epithelial cells or epithelial cells in general. Indeed both HeLa and Vero cells used in that study are of epithelial origin. Although virus production by NHBE cultures was lower than that reported for cultures of primary macrophages and T cells, it was within the range of values that have been reported for other non-immune primary cells [61,62].

We followed HIV infection over 50 days and observed that initially, p24 output declines rapidly but then stabilizes in the long-term. Most studies with epithelial cells have demonstrated these kinetics. One of the explanations for this decline in p24 could be due to a combined effect of failed integration events and death of infected cells. While all classes of multiply-spliced, singly spliced and unspliced viral mRNA transcripts can be observed from episomal proviral DNA [53,55], the proportion of these transcripts varies greatly with multiply splice transcripts being abundant while levels of unspliced transcripts being significantly lower [53,55]. This is due to low levels of Rev that are transcribed from unintegrated DNA [54]. Lack of rev limits nuclear export of full-length HIV transcripts, which in turn limits the levels of gag and env expression. Hence, we are able to observe low levels of p24 in the culture supernatants up to Day 50. RNA analysis by qRT-PCR Day 50 post-infection demonstrates cell-associated HIV RNA in both X4-infected and R5 infected NHBE ALI cultures. Upon entry, HIV undergoes reverse transcription to generate proviral DNA that is transcriptionally active as shown by qPCR analysis (Table 1) as well as using fluorescent HIV virus [43] (Fig 4A and 4B). In cells infected with the fluorescent virus, we observed significant cell death Day 12. Since the virus is env^-^, it is only capable of single round of infection suggesting that cell death could be due to expression of HIV genes. HIV-infected NHBE ALI cultures also secrete functionally active Tat that was able to transactivate Tat inducible GFP in co-cultured HEK-293 cells transfected with LTRshGFPpA. Together our observations demonstrate that NHBE ALI cultures can be infected with HIV and express viral proteins like Tat to suppress CFTR biogenesis and function. Our data agree with some observations of Brune et. al., [36] in that we also observe that HIV impairs lung epithelial integrity and we are able to detect viral RNA in infected cells. However we have also been able to demonstrate viral entry, reverse transcription and gene expression in primary human bronchial epithelial cells redifferentiated at the ALI. Our data differs from observations of Brune et al. in that they were unable to detect proviral DNA. This could be due to several differences in our experimental approach as well as culture conditions. Our NHBE ALI cultures are redifferentiated following a single expansion (Passage 1) after being isolated from the lung while Brune et. al. have primarily used commercially available cells which may have been expanded more than once before being provided to the consumer. Indeed we have observed that repeated passages alter the epithelium in subtle ways. We have observed decreased number of ciliated cells and lower CFTR activity when cells are passaged more than once (data not shown). We have used a different approach to detect proviral DNA. It is known that genomic DNA isolation methods and presence of high molecular weight genomic DNA can inhibit PCR [63] precluding detection of low levels of integrated or episomal viral DNA. We used a 2-step qPCR approach reported earlier to detect integrated HIV DNA [41]. For episomal DNA We have used plasmid miniprep isolation protocols to exclude genomic DNA. These methods enrich our DNA of interest before the qPCR step and improve PCR sensitivity [64,65].

Finally we demonstrate that HIV can also infect undifferentiated bronchial epithelial cells and impairs lung epithelial integrity and ciliogenesis. Most clinical studies have reported undetectable levels of HIV in patients on cART. Hence de novo infection of primary bronchial epithelial cells may be suppressed in presence of cART. However recurrent lung infections and other chronic inflammation associated with cigarette smoke or inhaled substance abuse can lead to recruitment of infected immune cells. These cells along with infected bronchial epithelial cells can serve as HIV reservoirs in the lung and lead to bursts of HIV Tat. In addition, these studies involve rigorous follow-up by research coordinators to minimize the incidence of missed doses. Hence it is unrealistic to assume that all patients will have undetectable levels of HIV all the time. The situation is exacerbated in people living with HIV who also abuse drugs as they intentionally miss cART doses when planning drug use [66,67]. Under these circumstances increased Tat expression or de novo infection of bronchial epithelial cells leading to disruption of bronchial epithelial integrity, decreased ciliogenesis and inhibition of CFTR biogenesis and function. In conclusion, this study demonstrates that HIV can infect NHBE ALI cultures and this results in suppression of components of the MCC apparatus by Tat mediated CFTR suppression, epithelial disruption and decreased ciliogenesis.

## Methods

### Cell culture

All experiments were performed with primary human bronchial epithelial cells, re-differentiated at the air–liquid interface (ALI). ALI cultures were prepared as described by Fulcher and Randall [68,69] and adapted by us [12,50]. Cells were isolated from appropriately consented donors whose lungs were found unsuitable for transplantation for reasons unrelated to airway disease and provided by the University of Miami Life Alliance Organ Recovery Agency. Since the material was obtained from deceased individuals with minor, de-identified information, its use does not constitute human subjects research as defined by CFR 46.102. A signed consent of each individual (or legal healthcare proxy) for donation of the lungs for research is on file with the Life Alliance Organ Recovery Organization covers research use of this material. Unless otherwise specified, experiments used cells from non-smokers to not confound the findings. The primary cultures undergo mucociliary differentiation at the ALI reproducing the *in vivo* morphology and key physiologic processes to regenerate the native bronchial epithelium *ex vivo* [68,69]. Epithelial cells from bronchial brushings were directly expanded at the ALI and used for experiments.

All methods were carried out in accordance with the approved guidelines. Additionally, stored epithelial cell brushes were used for this study. The material was obtained with clinical and demographic data and everything else was de-identified to protect confidentiality. All participants provided written informed consent. The study was approved by the University of Pittsburgh IRB.

### Electrophysiology experiments

Ussing chambers were used to determine CFTR activation [12]. The input resistance of each filter was measured by application of 1 mV bipolar pulses of 2s duration. Amiloride (10 μM) was added apically to eliminate Epithelial sodium channel (ENaC) influences. Following treatment with albuterol short circuit current (Isc) was allowed to stabilize. Experiments were terminated by adding CFTR_inh_172 (20 mM) to confirm that changes in I_SC_ were due to CFTR activation.

### Plasmids, virus strains and infections

Plasmid pRGH-WT (#12427), pBaL.01 (#11445) and pHXB2-env (# 1069) were obtained through the NIH AIDS reagent and reference program. The X4-tropic Viral strain HIV IIIB was a kind gift from Dr. John Rossi (City of Hope Medical Center, Duarte, CA). Unless otherwise mentioned, NHBE ALI cultures were infected with 5ngs p24 equivalent. RGH-WT virus was enveloped in HEK 293T cells by co-transfection with either R5-tropic (pBaL.01) or X4-tropic (pHXB2-env) envelope, pCMV Rev and pTatdsRed2 [47] plasmids. Supernatant was collected and the RGH-WT virus was purified as described by us earlier [12]. NHBE ALI cultures were infected with 10ngs equivalent of the R5- or X4-enveloped virus. GFP expression was followed by fluorescence microscopy.

### Detection of HIV proviral DNA

NHBE ALI cultures were infected with HIV IIIB (X4-tropic) or HIV BaL (R5-tropic) strains. Genomic DNA was isolated and integrated HIV DNA was determined by a 2-step qPCR method described by Brussel and Sonigo [41]. For episomal DNA, we used a modification of the method described by Buzon et. al. [42]. DNA was extracted using a Qiagen miniprep kit to isolate only episomal forms of DNA while excluding the genomic DNA. This bacterial plasmid DNA isolation method has been the most commonly used method for isolating 2-LTR circles from infected cells [64,65,70]. Eliminating high molecular weight nucleic acids from the sample, which might inhibit PCR [63], could enrich the episomal DNA and also improve PCR sensitivity. Copy numbers were determined by comparative Ct method using logarithmic dilutions of plasmid pNL4-3 to generate standard curves.

### Western blot analysis

Total protein was loaded onto a gel and run at 100V. Protein was transferred to PVDF membrane. Following transfer, blot was blocked in 5% milk then incubated in CD4 primary antibody (1: 500; Sigma-Aldrich), CXCR4 (1:1000; Thermo scientific) or CCR5 (1:1000; Thermo scientific). Blot was incubated in an anti-rabbit secondary antibody diluted to a concentration of 1:2500. Bands were detected in Chemidoc using Enhanced Chemiluminescence Reagents (ECL; Bio-Rad Laboratories).

### Data analysis

Unless otherwise stated, experiments were repeated three times using primary bronchial epithelial cells from at least three lung donors. Statistical significance was evaluated using unpaired t tests for two groups or ANOVA followed by Tukey Kramer honestly significant difference test for multiple comparisons as appropriate. P < 0.05 was considered significant.

## Supporting Information

S1 Fig NHBE ALI culture conditions do not demonstrate presence of immune cells. NHBE ALI culture cells immuno-stained with surface markers anti-EpCam (FCMAB264F, Milli-Mark) labeled with FITC or common lymphocyte marker, anti-CD45 (MA1-19569, Thermo Fisher Scientific) labeled with FITC. Negative unstained and isotype controls (MABC004F and MABC002F, Emd Millipore) were also analyzed. Data were acquired with Amnis^®^ FlowSight^®^ Imaging Flow Cytometer. Staining and image collection were carried out according to manufacture’s protocol. Images for compensation were collected with compensation beads (552843, BD Biosciences) labeled with the same antibody. Once images were captured, compensation and analysis was carried out with IDEAS^®^ image analysis software. From all events collected, first single cells were gated, then FITC+ cells were gated from histogram of intensity of FITC for 10^4^ or higher. The bar graphs represent the percentage of gated cells. **Panel A**: The graph represents % gated EpCam-isotype (4.45%), EpCam (51.89%), CD45-isotype (4.9%), and CD45 positive (0.95%) cells. **Panel B-E**: Representative histograms for NHBE cells used to calculate % of gated cells. **Panel F**: To check efficiency of staining and reactivity of antibodies, Peripheral Blood Mononuclear Cells (PBMCs) were labeled with anti-EpCam or anti-CD45 and % gated positive cells were calculated. EpCam shows no positive cells while CD45 shows a 78.2% positive cells. **Panel G-H**: Representative histograms for PBMC cells used to calculate % gated cells.
(TIFF)
