# Supplementary figures and images for: HIV Infects Bronchial Epithelium and Suppresses Components of the Mucociliary Clearance Apparatus
Source: PLoS One. 2017 Jan 6;12(1):e0169161. doi: 10.1371/journal.pone.0169161 (PMC5217953; doi:10.1371/journal.pone.0169161)

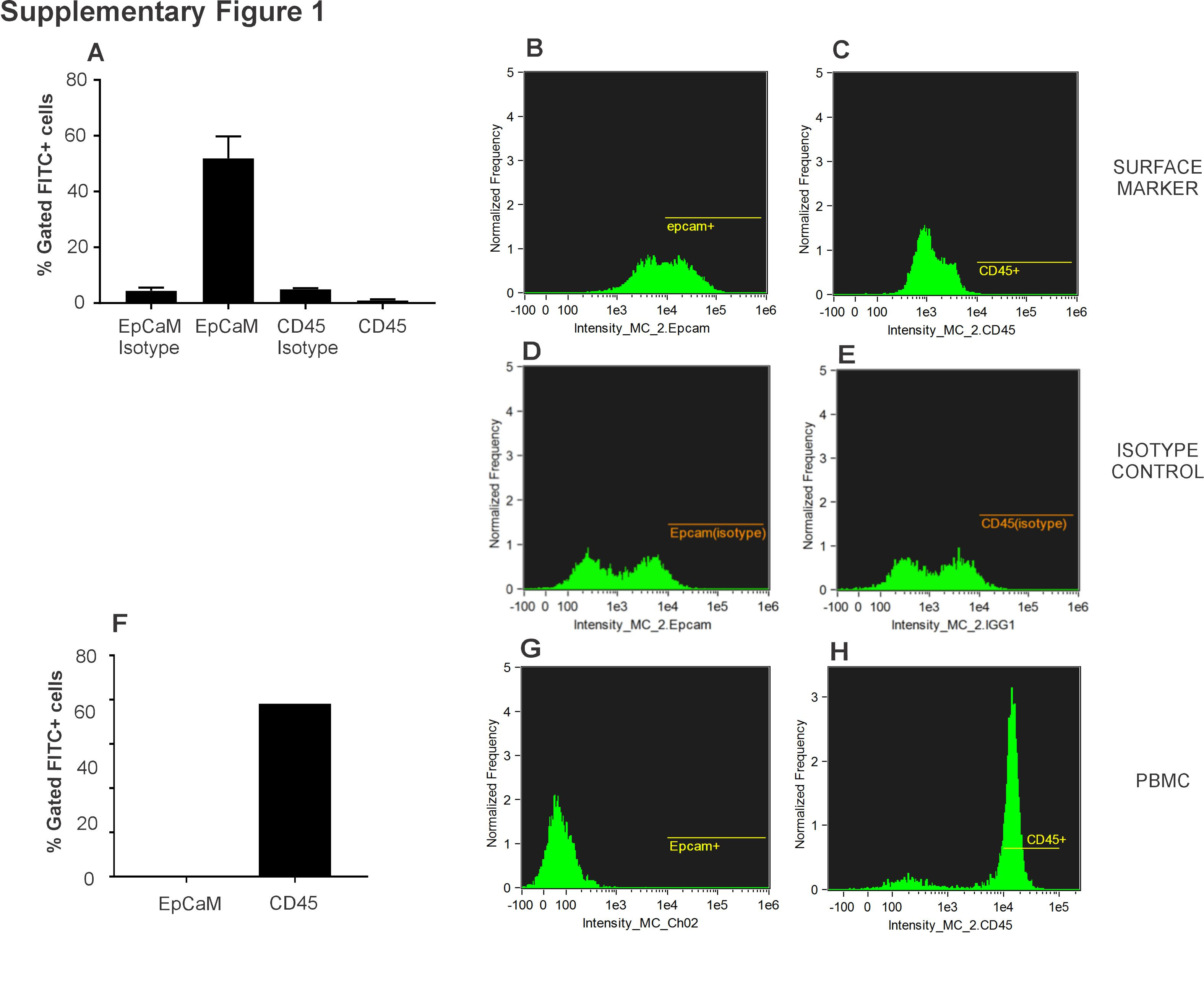

Supplement: S1 Fig — NHBE ALI culture cells immuno-stained with surface markers anti-EpCam (FCMAB264F, Milli-Mark) labeled with FITC or common lymphocyte marker, anti-CD45 (MA1-19569, Thermo Fisher Scientific) labeled with FITC. Negative unstained and isotype controls (MABC004F and MABC002F, Emd Millipore) were also analyzed. Data were acquired with Amnis® FlowSight® Imaging Flow Cytometer. Staining and image collection were carried out according to manufacture’s protocol. Images for compensation were collected with compensation beads (552843, BD Biosciences) labeled with the same antibody. Once images were captured, compensation and analysis was carried out with IDEAS® image analysis software. From all events collected, first single cells were gated, then FITC+ cells were gated from histogram of intensity of FITC for 104 or higher. The bar graphs represent the percentage of gated cells. Panel A: The graph represents % gated EpCam-isotype (4.45%), EpCam (51.89%), CD45-isotype (4.9%), and CD45 positive (0.95%) cells. Panel B-E: Representative histograms for NHBE cells used to calculate % of gated cells. Panel F: To check efficiency of staining and reactivity of antibodies, Peripheral Blood Mononuclear Cells (PBMCs) were labeled with anti-EpCam or anti-CD45 and % gated positive cells were calculated. EpCam shows no positive cells while CD45 shows a 78.2% positive cells. Panel G-H: Representative histograms for PBMC cells used to calculate % gated cells. (TIFF) [file pone.0169161.s001.tiff]
